# Supplementary figures and images for: A machine learning-enabled open biodata resource inventory from the scientific literature
Source: PLoS One. 2023 Nov 28;18(11):e0294812. doi: 10.1371/journal.pone.0294812 (PMC10684096; doi:10.1371/journal.pone.0294812)

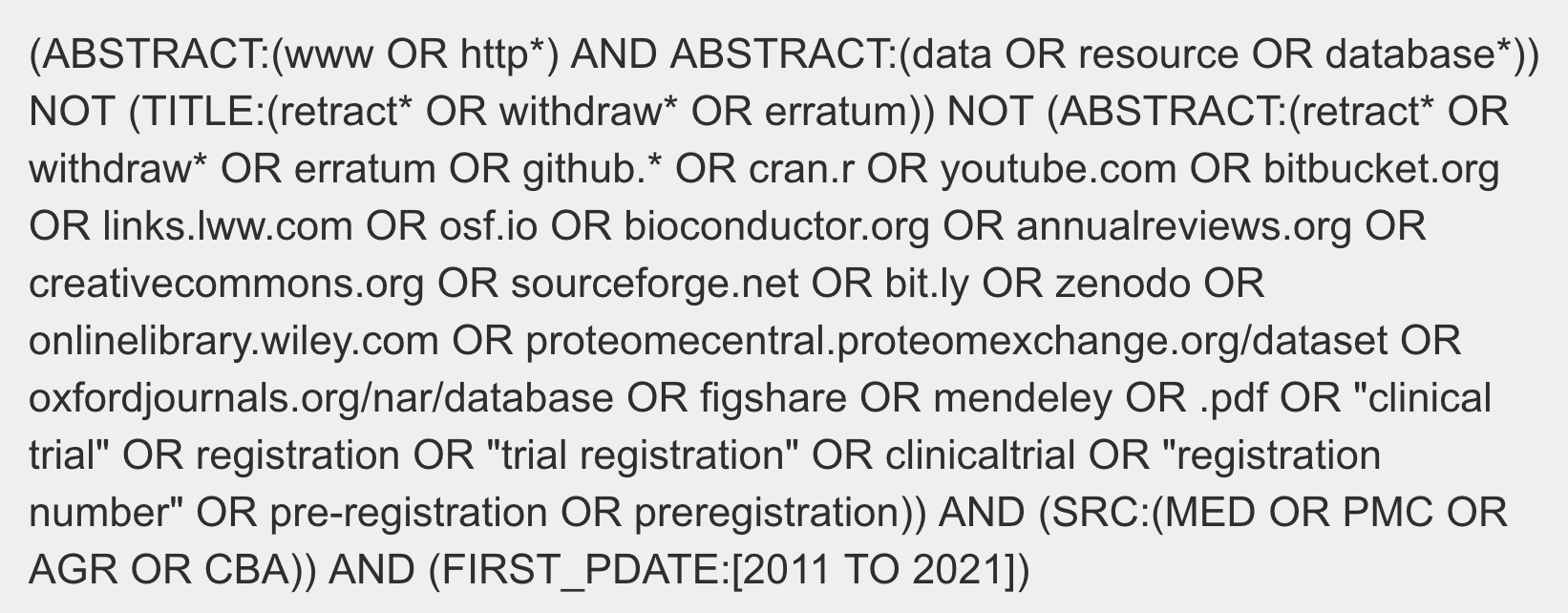

Supplement: S1 Fig — (TIF) [file pone.0294812.s001.tif]

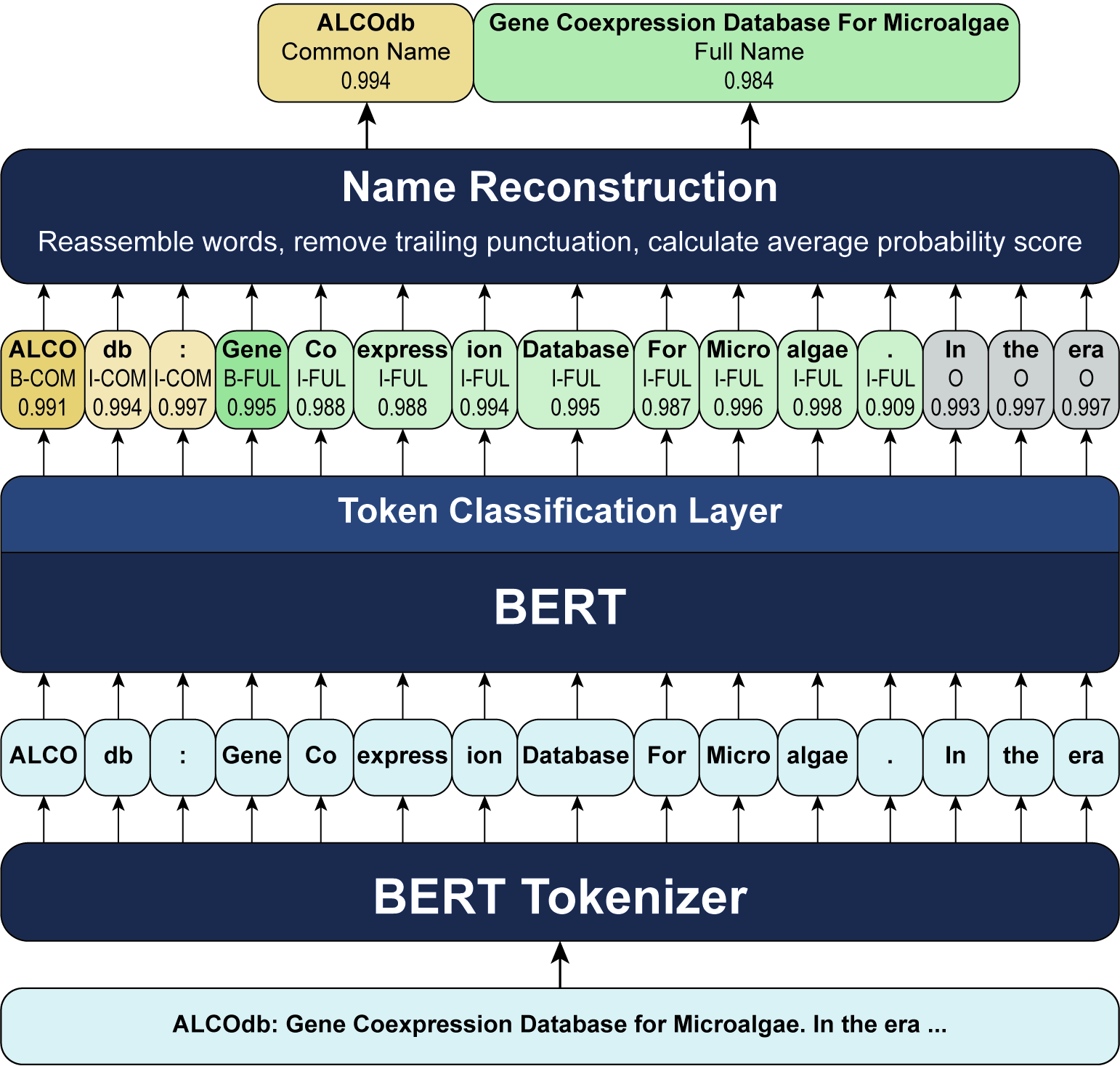

Supplement: S2 Fig — Process shows how the tokens are labeled using the BIO scheme and probability scores are output by the linear token classification layer of the BERT model. Tokens are then reassembled into words using the associated word indices (not shown), and the average probability score of the tokens is calculated. Trailing punctuation is removed from predicted resource names. (TIF) [file pone.0294812.s002.tif]

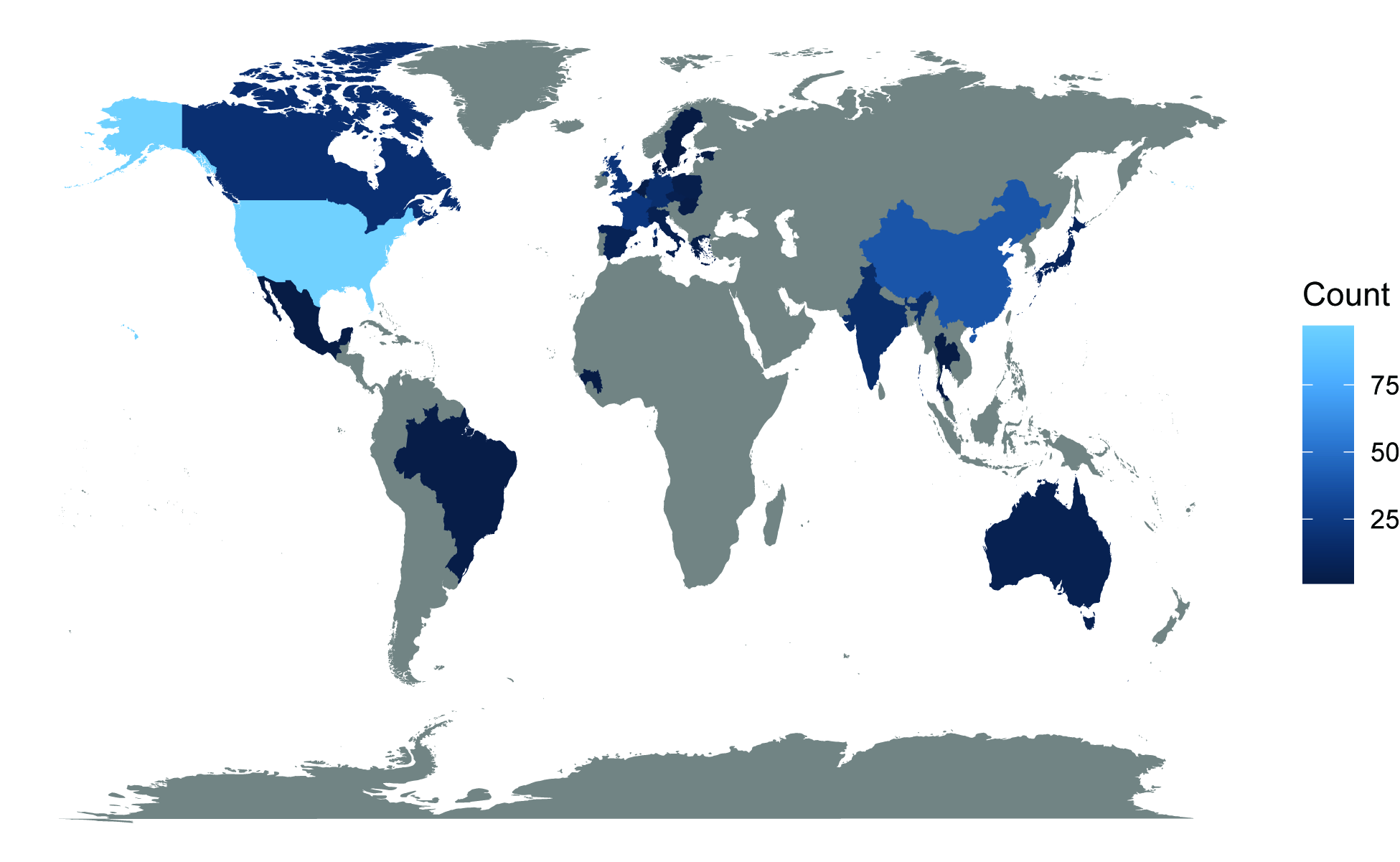

Supplement: S3 Fig — Choropleth map shows URL host IP address countries based on matches to ISO-3166-1 country names or Alpha-3 codes. Color is scaled to the number of times that country’s name appeared as a host IP address location. Figure was created using the R ggplot2 package which obtains map data from Natural Earth [71], which is in the public domain. (TIF) [file pone.0294812.s003.tif]

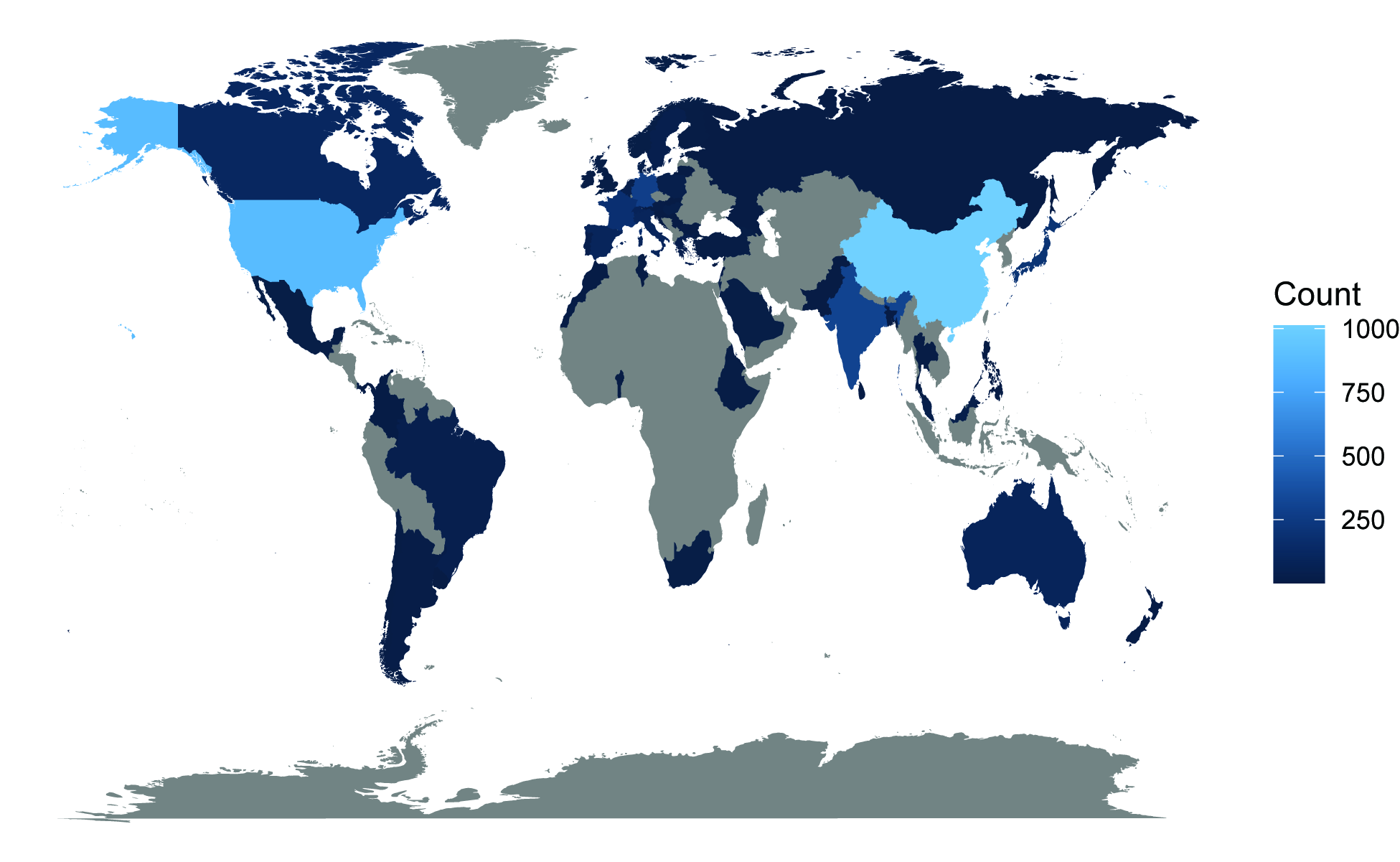

Supplement: S4 Fig — Choropleth map shows author affiliation countries based on matches to ISO-3166-1 country names or Alpha-3 codes. Color is scaled to the number of times that country’s name appeared in the author affiliations across all articles in the inventory. Figure was created using the R ggplot2 package which obtains map data from Natural Earth [71], which is in the public domain. (TIF) [file pone.0294812.s004.tif]
