# Supplementary material for: A machine learning-enabled open biodata resource inventory from the scientific literature
Source: PLoS One. 2023 Nov 28;18(11):e0294812. doi: 10.1371/journal.pone.0294812 (PMC10684096; doi:10.1371/journal.pone.0294812)
Supplement: S1 Table — (PDF) [file pone.0294812.s005.pdf]

S1 Table. Definitions consulted for "Life Sciences Biodata."

Source	Definition found	URL
Computer Retrieval of Information on Scientific Projects Thesaurus	CRISP:Biology<br>Definition: "science concerned with the phenomena of life and living organisms"	https://web.archive.org/web/20230106212709/https://bioportal.bioontology.org/ontologies/CRISP?p=classes&conceptid=0418-4282
National Cancer Institute Thesaurus	NCIT:Basic Research<br>Definition: "Fundamental research designed to obtain or increase general scientific knowledge."	https://web.archive.org/web/20230106212857/https://bioportal.bioontology.org/ontologies/NCIT?p=classes&conceptid=http%3A%2F%2Fncicb.nci.nih.gov%2Fxml%2Fowl%2FEVS%2FThesaurus.owl%23C15714
Wikipedia	List of Life Sciences<br>"branches of science that involve the scientific study of life – such as microorganisms, plants, and animals including human beings"	https://web.archive.org/web/20211023011543/https://en.wikipedia.org/wiki/List_of_life_sciences
Wikipedia	Basic Research<br>"type of scientific research with the aim of improving scientific theories for better understanding and prediction of natural or other phenomena"	https://web.archive.org/web/20211019010200/https://en.wikipedia.org/wiki/Basic_research
