# Supplementary material for: A machine learning-enabled open biodata resource inventory from the scientific literature
Source: PLoS One. 2023 Nov 28;18(11):e0294812. doi: 10.1371/journal.pone.0294812 (PMC10684096; doi:10.1371/journal.pone.0294812)
Supplement: S2 Table — (PDF) [file pone.0294812.s006.pdf]

S2 Table. Definitions consulted for "Biodata Resource."

Source	Definition found	URL
DCAT	dcat:DataService<br>Definition: A collection of operations that provides access to one or more datasets or data processing functions.	https://web.archive.org/web/20221225101619/https://www.w3.org/TR/vocab-dcat-3/#Class:Data_Service
Biomedical Resource Ontology	BRO:Data_Resource<br>Definition: A resource that provides individual facts, statistics or items of information.	https://web.archive.org/web/20230106213856/https://bioportal.bioontology.org/ontologies/BRO/?p=classes&conceptid=http%3A%2F%2Fbioontology.org%2Fontologies%2FBiomedicalResourceOntology.owl%23Data_Resource
DOE	"PuRe Data Resources are data repositories, knowledge bases, and analysis platforms that are sponsored by the Office of Science."	https://web.archive.org/web/20220608144146/https://science.osti.gov/Initiatives/PuRe-Data/Frequently-Asked-Questions
NIH	"... defines data repositories as data resources that store, organize, validate, and make accessible the core data related to a particular system or systems. For example, core data might include genome, transcriptome, and protein sequences for one or more organisms. Knowledgebases are defined as resources that accumulate, organize, and link growing bodies of information related to core datasets. They are resources that may contain, for example, information about gene-expression patterns, splicing variants, localization, and protein-protein interactions and pathway networks related to an organism or set of organisms."	https://web.archive.org/web/20220913053932/https://datascience.nih.gov/sites/default/files/Metrics-Report-2021-Sep15-508.pdf
re3data.org	"A research data repository is a subtype of a sustainable information infrastructure which provides long-term storage and access to research data that is the basis for a scholarly publication. Research data means information objects generated by scholarly projects for example through experiments, measurements, surveys or interviews."	https://web.archive.org/web/20211021235443/https://www.re3data.org/suggest
ELIXIR Core Data Resources	"are of fundamental importance to the wider life-science community and the long-term preservation of biological data. They provide complete collections of generic value to life-science, are considered an authority in their field	https://doi.org/10.12688/f1000research.9656.2

	with respect to one or more characteristics, and show high levels of scientific quality and service"	
