# Supplementary material for: A machine learning-enabled open biodata resource inventory from the scientific literature
Source: PLoS One. 2023 Nov 28;18(11):e0294812. doi: 10.1371/journal.pone.0294812 (PMC10684096; doi:10.1371/journal.pone.0294812)
Supplement: S3 Table — (PDF) [file pone.0294812.s007.pdf]

S3 Table. APIs used.

Source	Purpose	Documentation
Europe PMC	article corpus	https://web.archive.org/web/20220602202816/https://EuropePMC.org/docs/EBI_Europe_PMC_Web_Service_Reference.pdf
Wayback Machine	URL archive	https://web.archive.org/web/20230106002819/https://archive.org/help/wayback_api.php
re3data.org	resource comparison	https://web.archive.org/web/20230106154142/https://www.re3data.org/api/doc
FAIRsharing	resource comparison	https://fairsharing.org/API_doc
ipinfo	geolocation of IP addresses	https://web.archive.org/web/20230101155943/https://ipinfo.io/developers
ip-api	geolocation of IP addresses	https://web.archive.org/web/20221210042446/https://ip-api.com/docs
