# Supplementary material for: A machine learning-enabled open biodata resource inventory from the scientific literature
Source: PLoS One. 2023 Nov 28;18(11):e0294812. doi: 10.1371/journal.pone.0294812 (PMC10684096; doi:10.1371/journal.pone.0294812)
Supplement: S4 Table — (PDF) [file pone.0294812.s008.pdf]

S4 Table. Hyperparameters used for model fine-tuning for article classification and NER tasks.

Model	Batch Size	Learning Rate	Weight Decay	Learning Rate Scheduler
BERT	16	3e-5	0	False
BioBERT	16	3e-5	0	False
BioELECTRA	16	5e-5	0	True
BioELECTRA-PMC	32	5e-5	0	True
BioMed-RoBERTa	16	2e-5	0	False
BioMed-RoBERTa-CP	16	2e-5	0	False
BioMed-RoBERTa-RCT	16	2e-5	0	False
BlueBERT	16	3e-5	0	True
BlueBERT-MIMIC-III	32	3e-5	0	False
ELECTRAMed	16	5e-5	0	True
PubMedBERT	16	3e-5	0	True
PubMedBERT-Full	32	3e-5	0	True
SapBERT	16	2e-5	0.01	False
SapBERT-Mean	32	2e-5	0.01	False
SciBERT	16	3e-5	0	False
