# Supplementary material for: A machine learning-enabled open biodata resource inventory from the scientific literature
Source: PLoS One. 2023 Nov 28;18(11):e0294812. doi: 10.1371/journal.pone.0294812 (PMC10684096; doi:10.1371/journal.pone.0294812)
Supplement: S5 Table — Performance metrics are shown for both the validation and test sets. Models are arranged in decreasing order of precision on the validation set, which was used for model selection. (PDF) [file pone.0294812.s009.pdf]

S5 Table. Article classification model performance.

Performance metrics are shown for both the validation and test sets. Models are arranged in decreasing order of precision on the validation set, which was used for model selection.

	Validation Set			Test Set		
**Model**	**F1-score**	**Precision**	**Recall**	**F1-score**	**Precision**	**Recall**
BioMed-RoBERTa-RCT[1]	0.849	0.939	0.775	0.821	0.975	0.709
BioMed-RoBERTa-CP	0.800	0.933	0.700	0.791	1.000	0.655
SciBERT	0.800	0.933	0.700	0.791	1.000	0.655
BioBERT	0.783	0.931	0.675	0.821	0.975	0.709
BERT	0.708	0.920	0.575	0.721	1.000	0.564
BioELECTRA-PMC	0.667	0.913	0.525	0.750	1.000	0.600
BioMed-RoBERTa	0.838	0.912	0.775	0.900	1.000	0.818
SapBERT-Mean	0.838	0.912	0.775	0.857	0.977	0.764
BioELECTRA	0.806	0.906	0.725	0.800	0.950	0.691
PubMedBERT	0.806	0.906	0.725	0.854	1.000	0.745
SapBERT[2]	0.886	0.897	0.875	0.874	0.938	0.818
ELECTRAMed	0.846	0.868	0.825	0.871	0.957	0.800
PubMedBERT-Full	0.846	0.868	0.825	0.866	1.000	0.764
BlueBERT	0.773	0.829	0.725	0.860	0.956	0.782
BlueBERT-MIMIC-III	0.773	0.829	0.725	0.832	0.913	0.764

[1]Model with highest precision on validation set that was used to generate final inventory
[2]Model with highest *F*1-score on validation set that was used during mid-project evaluation
