# Supplementary material for: A machine learning-enabled open biodata resource inventory from the scientific literature
Source: PLoS One. 2023 Nov 28;18(11):e0294812. doi: 10.1371/journal.pone.0294812 (PMC10684096; doi:10.1371/journal.pone.0294812)
Supplement: S6 Table — Performance metrics are shown for both the validation and test sets. Models are arranged in decreasing order of F1 score on the validation set, which was used for model selection. (PDF) [file pone.0294812.s010.pdf]

S6 Table. NER model performance.

Performance metrics are shown for both the validation and test sets. Models are arranged in decreasing order of *F*1 score on the validation set, which was used for model selection.

	Validation Set			Test Set		
**Model**	**F1-score**	**Precision**	**Recall**	**F1-score**	**Precision**	**Recall**
BioMed-RoBERTa-RCT[1]	0.683	0.674	0.693	0.717	0.689	0.748
BioMed-RoBERTa	0.660	0.685	0.638	0.688	0.681	0.695
SapBERT-Mean	0.651	0.738	0.583	0.651	0.678	0.626
PubMedBERT-Full	0.648	0.717	0.592	0.688	0.736	0.646
SapBERT	0.646	0.733	0.578	0.629	0.674	0.589
SciBERT	0.646	0.680	0.615	0.673	0.656	0.691
BERT	0.644	0.682	0.610	0.703	0.699	0.707
PubMedBERT	0.642	0.620	0.665	0.652	0.638	0.667
BioMed-RoBERTa-CP	0.632	0.646	0.619	0.684	0.686	0.683
BioBERT	0.629	0.644	0.615	0.671	0.693	0.650
BioELECTRA-PMC	0.606	0.649	0.569	0.658	0.675	0.642
BlueBERT	0.606	0.654	0.564	0.643	0.665	0.622
BioELECTRA	0.585	0.598	0.573	0.613	0.605	0.622
BlueBERT-MIMIC-III	0.573	0.608	0.541	0.585	0.567	0.606
ELECTRAMed	0.567	0.647	0.505	0.651	0.670	0.634

[1]Model with highest *F*1-score on validation set that was used during mid-project evaluation and generation of the final inventory
