# Supplementary material for: A machine learning-enabled open biodata resource inventory from the scientific literature
Source: PLoS One. 2023 Nov 28;18(11):e0294812. doi: 10.1371/journal.pone.0294812 (PMC10684096; doi:10.1371/journal.pone.0294812)
Supplement: S7 Table — (PDF) [file pone.0294812.s011.pdf]

Table S7. Open science products.

Type	Name	GitHub (living)	Zenodo (archive)	Hugging Face Hub	protocols.io
data	exact ePMC query output (title-abstract)	https://github.com/globalbiodata/inventory_2022/blob/main/data/epmc_query_results_2022.csv	https://zenodo.org/doi/10.5281/zenodo.10105161	n/a	n/a
data	Article classification training data	https://github.com/globalbiodata/inventory_2022/blob/main/data/manual_classifications.csv	https://zenodo.org/doi/10.5281/zenodo.10105161	n/a	n/a
data	NER training data	https://github.com/globalbiodata/inventory_2022/blob/main/data/manual_ner_extraction.csv	https://zenodo.org/doi/10.5281/zenodo.10105161	n/a	n/a
data	manually reviewed inventory	https://github.com/globalbiodata/inventory_2022/blob/main/data/manually_reviewed_inventory.csv	https://zenodo.org/doi/10.5281/zenodo.10105161	n/a	n/a
data	final inventory	https://github.com/globalbiodata/inventory_2022/blob/main/data/final_inventory_2022.csv	https://zenodo.org/doi/10.5281/zenodo.10105947	n/a	n/a
code	Python scripts and modules	https://github.com/globalbiodata/inventory_2022/tree/main/src	https://zenodo.org/doi/10.5281/zenodo.10105161	n/a	n/a
code	Snakemake workflows	https://github.com/globalbiodata/inventory_2022/tree/main/snakemake	https://zenodo.org/doi/10.5281/zenodo.10105161	n/a	n/a
code	ipython notebook for reproducing the original results	https://github.com/globalbiodata/inventory_2022/blob/main/running_pipeline.ipynb	https://zenodo.org/doi/10.5281/zenodo.10105161	n/a	n/a
code	ipython notebook for inventory update	https://github.com/globalbiodata/inventory_2022/blob/main/updating_inventory.ipynb	https://zenodo.org/doi/10.5281/zenodo.10105161	n/a	n/a
configurations	query	https://github.com/globalbiodata/inventory_2022/blob/main/config/query.txt	https://zenodo.org/doi/10.5281/zenodo.10105161	n/a	n/a

configurations	model fine-tuning parameters	https://github.com/globalbiodata/inventory_2022/blob/main/config/models_info.tsv	https://zenodo.org/doi/10.5281/zenodo.10105161	n/a	n/a
configurations	Snakemake and directory structure configurations	https://github.com/globalbiodata/inventory_2022/blob/main/config/train_predict.yml	https://zenodo.org/doi/10.5281/zenodo.10105161	n/a	n/a
configurations	Python code formatting and linting configurations	https://github.com/globalbiodata/inventory_2022/blob/main/config/.pylintrc	https://zenodo.org/doi/10.5281/zenodo.10105161	n/a	n/a
models	fine tuned BERT models	n/a	n/a	https://huggingface.co/globalbiodata/inventory/tree/main	n/a
documentation	README (main)	https://github.com/globalbiodata/inventory_2022/blob/main/README.md	https://zenodo.org/doi/10.5281/zenodo.10105161	n/a	n/a
documentation	Google Colab protocol	n/a	n/a	n/a	https://www.protocols.io/view/set-up-biodata-resource-inventory-in-google-colab-5jyl89o36v2w/v1
documentation	open science implementation plan	n/a	https://doi.org/10.5281/zenodo.7392518	n/a	n/a
documentation	curation guide for selective review	n/a	https://doi.org/10.5281/zenodo.7768363	n/a	n/a
documentation	use case article pre-print	n/a	https://doi.org/10.5281/zenodo.7767793	n/a	n/a
documentation	full article pre-print	n/a	https://doi.org/10.5281/zenodo.7768415	n/a	n/a
