# Supplementary material for: A machine learning-enabled open biodata resource inventory from the scientific literature
Source: PLoS One. 2023 Nov 28;18(11):e0294812. doi: 10.1371/journal.pone.0294812 (PMC10684096; doi:10.1371/journal.pone.0294812)
Supplement: Attachment — Submitted filename: PONE-D-23-16263 Response to Reviewers.pdf [file pone.0294812.s012.pdf]

September 14, 2023

Subject: Response to Reviewers for Manuscript No. PONE-D-23-16263

Dear Editor Bernasconi,

As indicated in our cover letter for the revision, points related to PLOS requirements are included there.

We appreciate reviewers' careful and valuable comments, and here we provide inline responses to those comments, indicating where changes have been made to the revised manuscript or our rationale when we declined to make changes. For each point, our response is provided in green and begins with "**Our Response:**". For brevity, we have removed some details in each point (e.g. links to PLOS ONE's style guides or points which did not require action on our part such as rigor of statistical analysis). Our responses follow the same order as in the decision letter received.

Reviewer Prompts/Comments

1. Is the manuscript technically sound, and do the data support the conclusions?

    Reviewer #1: Yes
    Reviewer #2: Partly
    Reviewer #3: Yes

    Our Response: Actions taken to address Reviewer 2's "Partly" are below.

2. Has the statistical analysis been performed appropriately and rigorously?

    Reviewer #1: Yes
    Reviewer #2: N/A
    Reviewer #3: Yes

    Our Response: No action needed.

3. Have the authors made all data underlying the findings in their manuscript fully available?

    Reviewer #1: Yes
    Reviewer #2: Yes
    Reviewer #3: Yes

    Our Response: No action needed.

4. Is the manuscript presented in an intelligible fashion and written in standard English?

Reviewer #1: Yes
Reviewer #2: Yes
Reviewer #3: Yes

Our Response: No action needed.

5. Review Comments to the Author

Reviewer #1:

General appreciation: The ML pipelines for the identification of biodata resources were diligently constructed with several verifications along the road. The results have been validated in several manners. The publication is mostly about the method, not about the resulting inventory (which may be at least as interesting).

Our Response: We are gratified to read these positive comments. We did choose to focus this publication on the method itself, but we agree that the inventory is just as interesting and have commented below on potential follow-up work.

- Error to be corrected: Line 489: the figure says ESTHER, not ESTER

  Our Response: This error has been corrected. Thank you for the keen eyes.

Elements that would be valuable to amend are:

- Lines 672-673: describes the "WHY?" of this inventory. However, this is not well explained nor convincing. The Global Biodata Coalition is about sustaining important databases worldwide and this publication aims to establish a method to describe the background landscape of data resources. Why is this useful? Why do we need the inventory and/or what will be done with it? How does it feed into the work of the Coalition?

  Our Response: We appreciate this feedback and have added a paragraph to make the purpose and value of this work more explicit in Section 4. Discussion.

- There is no mentioning about the language of the underlying data resources (this is on a corpus of English-language publications). This seems nevertheless relevant for a global inventory.

  Our Response: Thank you for catching this omission as this is very important to note. We edited the methods section to state that the Europe PMC API was accessed to obtain English-language articles, and we further revised to include this point in the discussion as well.

- Section 3.5.3: The section on the comparison with re3data.org and FAIRSHARING could be worked out more diligently. The overlap is less than 20%, leading the authors to state that both methods are complementary. Being

complementary implies that their purpose is similar and that the 3 inventories will feed into the work of the Global Biodata Coalition. (This again points to the WHY? of the work as they can only be complementary if the aim is similar.) There are alternative explanations possible that are not addressed: The underlying collections of resources are not at all comparable and therefore both inventories are not valid benchmarks. Or the method described by the authors is missing a large part of the resources. Further clarification is therefore needed. NAR would have been a useful source for a verification. Is there any other way to use the NAR data for verification through a human, manual check as the FAIRSHARING/re3data.org verification has not allowed any firm conclusions?

Our Response: These are all terrific points, and we revised the text in the discussion to clarify. As the reviewer points out, there are many possible explanations. From the preliminary work presented, we know that carefully teasing apart the possibilities would certainly require a substantial amount of human curation and would constitute another study entirely. Our goal with this publication was to gauge reception of and interest in the inventory and our methods for assembling it. So while a more detailed analysis is out of the scope of this publication, which is already quite lengthy and detailed (too lengthy according to another reviewer), we absolutely agree that it is warranted and are very happy to hear there is interest in additional analyses.

- Section 3.4 about URL testing: 29% of the URLs did not resolve. There would be another 6.5% false positives. We know that many data resources are archived over time or disappear. Is this number of about 35% of data resources unavailable within what is expected based on the literature?

  Our Response: It's roughly within expectations, although on the high side when adding in the false positive estimate. Previous studies reported ~27%, noting that it's highly time dependent and also appears related to popularity (as determined by citations). We haven't assessed the up/down breakdown by year or citation, but those values are included in the inventory if anyone wants to investigate. We added text to this extent in this section.

Reviewer #2:

Overall, the authors make a compelling case for the need for a pipeline to find biodata resources automatically based on the reliance of biological science on such resources. The focus on open science is also very valuable for future use and updates of such a repository of biodata resources. At the same time, the article needs more details on specific design choices as well as more exploration of the resulting repository to provide evidence for the authors' claims. Further, from a writing perspective, some of the sentences are too long and some content needs to be reorganized into different article sections. In general though, the idea is interesting and warrants publication after revisions. See below for more detailed revisions (in order of the article).

Our Response: In addition to the specific response below, we'd sincerely thank this reviewer for their careful reading and thoughtful consideration of our work. It is gratifying to see appreciation for our efforts toward open science and reproducibility. Their detailed suggestions and comments have been very helpful in shaping this manuscript into a more readable and useful addition to the literature.

The reviewer did have many suggestions for restructuring that we considered in all cases and fully or partially implemented in many. We are very sympathetic to the reviewer's preference for a crisper and more linear presentation. However, we found this paper particularly difficult to write, in part because of the interactive nature of the process and the mid-project evaluation. Because of this, we deliberately sought out and followed PLOS One's guidance for structuring a discussion as we prepared the manuscript. This guidance (https://plos.org/resource/how-to-write-conclusions/) encourages authors to focus on key findings. We have reworked the paper considerably in light of the reviewer's comments, but we did not revise the discussion to systematically cover *all* findings as the reviewer seemed to request. We also note that the other two reviewers did not seem to mind the structure we presented, with one specifically stating they found the manuscript well written and structured.

Major Revisions:

- *1. There are many long sentences. Example, in the introduction the last sentence of the 2nd paragraph is very long and confusing ("However, and again unlike physical sciences,..."). Another example is the last sentence of the first paragraph in the methods ("while there are limitations...").*

  Our Response: We have made edits to reduce long sentences and improve the readability of our manuscript throughout, including the two examples given.

- *2. One of the discussion points in the end is that the list of data repositories found can be used to contact the owners of the data repositories to register them. This point though is buried in the introduction in the paragraph that starts "Blair et al". More details about registering databases is necessary to foreshadow this finding and the importance of it in general.*

  Our Response: This is a very helpful suggestion.Text has been added.

- *3. The query used to retrieve publications that contain a data repository is not well explained. It is mentioned that they "developed a targeted query", but it is not clear to me how that was developed. More details appear later about the issue with other types of URLs. While later Figure S1 is pointed to with the query, it is not explained (data resources page 9). In terms of the query itself, the term "repository" is notably missing. Also "knowledge-base". Further, for training purposes on the article classifier, it is interesting that there are no true negative articles in the dataset. Meaning random articles that do not talk about data resources at all. On some level, one could think of any new article and classifying*

it as having a data resource or not immediately without the targeted query each time. Also, new words for data resources may appear and so would the query be updated to accommodate for this? In general, more details on the corpus construction would be beneficial.

Our Response: We've added text on query development to the method section. We've also used "repository" as an example term which is used in different, but related, contexts that creates other challenges (e.g. conflation with code repositories and especially those associated with data analysis servers). We explicitly designed the pipeline so that a user-provided query can be provided. We're happy to make that feature more explicit, so this text was added there, too.

Regarding the "no true negatives" we see this point in theory, but in practice it did not work that way entirely. Although we developed a query to enrich the set with articles describing a resource, from our curation efforts we know that it didn't entirely exclude those that didn't. These were not always random articles but still true negatives for our purposes. For example, articles that described use of data and then referenced the URL for that data source in the abstract were common. Some others were quite left field. For example, computer science articles that included "WWW" and "data" in the abstract. To train the classifier on all articles certainly could be an interesting follow-up effort but a substantial one both in terms of labeling data and computation. For this effort, what we hoped to learn was if this strategy was going to help us generate a reasonably accurate inventory and what all would be needed post-NLP to make that inventory of use to those who we believed would be interested in it.

- *4. One of the main evaluations of this work is a comparison to other data inventory organizations like re3data and FAIRsharing. In the methods, there needs to be an acknowledgement of the different possibilities with this comparison (i.e., what does a large overlap mean vs. a small overlap) and that in general, these inventories do not include all repositories. This should foreshadow section 3.5.3.*

  Our Response: This is a good point that may not be obvious to everyone. We have added text to address this observation in the methods, which now also contains additional details suggested in other points made by the reviewer.

- *5. There needs to be a clear distinction between the methods and results sections. For example, under section 2.5.1, the last few sentences of the first paragraph are results. The methods should state that inter-annotator agreement was calculated and how it was calculated and that an error analysis was conducted. The results should be in results. It is not clear to me also where discussion fits into this. I think a separate section would be helpful. Without this distinction there is a fair amount of repetitive content.*

Our Response: We are deeply sympathetic to this comment. When we tried a stricter delineation, the methods seemed disjointed without some allusion to what was learned and why we did x, y, z. We have taken a fresh look throughout though and pointed readers to results instead in more places, for example in the IAA. We have also revised to remove redundant content wherever possible. Please see our opening response regarding the discussion section.

- *6. A discussion on the interplay between the two tasks (article classification and NER) needs to be discussed. Errors will propagate between tasks.*

  Our Response: This is a good point, and we revised the text in the discussion to touch on this. In particular, we find that since the article classification model had such high precision, the risk of error propagation is minimized, but it is certainly true that the names of things which are not biodata resources may still be predicted by the NER model.

- *7. It is mentioned that the best algorithm is chosen based on the validation set and I am unclear why this is not on the test set? This needs to be further clarified.*

  Our Response: We are following the convention for ML model selection as can be found in Wulf et al., 2022 (now cited in the manuscript) which seemed most appropriate to our use case. We have revised the text in the methods section 2.5.1 when introducing the training data sets.

- *8. Precision was chosen over F1 for article classification. This is somewhat explained later and clearly recall is low based on the results. However, in methods this needs to be explained. Ideally, the motivation for focusing on different metrics exists in the introduction. (page 15) Some explanation exists on page 25 with "we explored putting greater weight on precision than F1…".*

  Our Response: We've added text to the introduction and in Section 2.5.3. for the article classification task.

- *9. Section 2.9 needs more details on the analyses performed on the inventory itself. The results mention a lot of methods around this that should be moved here. Further, other types of analyses like the publication dates of repositories, the number of articles each repository has, etc. would continue to highlight the value of this work.*

  Our Response: We've added more detail, rearranged the results and methods some, and also moved supplemental material into newly created subsections in Section 2.9. With regard to additional analyses, our goal with this publication was to gauge reception of and interest in the inventory and our methods for assembling it. The preliminary analyses were intended to highlight what could be done with the inventory since there are many, many avenues for further analyses. In fact, the ideas suggested here are one direction and another reviewer suggested an entirely different direction. This article is already quite lengthy and

complicated (another reviewer suggested we shorten it), so we believe more detailed analyses are out of the scope for this publication. However, we absolutely agree that it is warranted and are very happy to hear there is interest in additional analyses.

- *10. The NLP results are interesting (section 3.2) and need to be discussed more. The precision is very high for the article classification and it is not clear to me why that is true. Also the test and validation sets disagree on the best performing algorithm. The test set has very high scores also. A mention of this here would be good to foreshadow the issue in data that is mentioned later.*

  Our Response: We have added more text to the discussion section to address the high precision of the article classification model on the test set, the lower performance of the NER model, and potential explanations for differences in performance. While it is true that the test and validation sets disagree, the model is selected based on the validation set, otherwise researchers risk biasing their selection to the test set results, rendering them no longer a good indicator of how the model will perform on unseen data.

- *11. The URL information should be introduced earlier (section 3.4) with the background on the analysis of resolving URLs and issues previous research has had. A discussion section is missing and so it is confusing that results also have discussion in it. Sections 3.5.1 and 3.5.2 are much more discussion based then just results. In 3.5.2, future work is mentioned that should be in a discussion section.*

  Our Response: We've moved text out of supplements into Section 3.4 to try address the reviewer's comments, but it is not clear to us where we would have introduced the URL information earlier so we respectfully decline this suggestion. Please see the opening response above in regards to the discussion.

- *12. The overlap comparison (section 3.5.3) needs more details in it and a further analysis. The authors speculate at the differences but if the authors want to claim that their work complements the other work, then it needs to be clear how. Should all the repositories the authors found be added into the other inventories? Do they have different purposes? This is especially crucial when they claim they could reach out to authors to add their databases.*

  Our Response: Details have been added to the methods section to describe the registries, their selection criteria, and our access, filtering, and cleaning processes. This was an oversight on our part not to include this information, and we appreciate that the reviewer caught this omission. Additionally, we revised the discussion to state more clearly how our work may complement existing registries without saying that it does already and to emphasize that additional work must be done. To the best of our knowledge, neither re3data or FAIRsharing's processes for identifying resources are assisted by ML, so it

seems to us that the method itself is one point of potential complementarity. It is not clear yet the *extent* to which the inventory will complement the actual collections of these registries, so we see how this may have been too premature to state. This has been revised as well. As far as additional analyses to get at that, we know from the preliminary work presented that this will certainly require a substantial amount of human curation and would constitute another study entirely. Similarly, "*Should all the repositories the authors found be added into the other inventories?"* is a terrific question, but it's not a question we can answer in the present study nor do we think we should try to address it alone. We're simply noting that since we have corresponding author information, at least such outreach is now a possibility.

<u>Minor Revisions:</u>

- 1. A citation is missing for Blair et al on page 5.

  Our Response: The citation has been added. Thank you for the keen eyes.

- 2. Does the working definition of biodata include the data in biomedical knowledge-bases or ontologies?

  Our Response: Computer Retrieval of Information on Scientific Projects Thesaurus (CRISP), National Cancer Institute Thesaurus (NCIT), Data Catalog Vocabulary (DCAT), and the Biomedical Resource Ontology (BRO) were all consulted as we put together definitions (see Tables S1 and S2). Or is the reviewer asking if these would meet the criteria for a biodata resource in our study? If so, yes, although we did not check to see if any of these surfaced.

- 3. It is mentioned that models are trained for max of 10 epochs. Why?

  Our Response: This was based on general convention as a starting point. The text has been revised to state this.

- 4. Classically the BIO/IBO/IOB scheme is B = beginning, I = inside, and O = outside. The otherwise is interesting here (page 15)

  Our Response: This was a typo that we've corrected. Thank you for the careful reading.

- 5. The mid-project evaluation is interesting and creates a very high probability threshold. The exact threshold is more results and so more explanation of where the threshold came from would be beneficial.

  Our Response: This comment seems to make our point above about the challenge between the methods and results section for this article. *Is* it "more results"? When we did that originally, it seemed awkward that it wasn't in the methods. We currently have an explicit parenthetical to reference readers to the results in that methods section, so we believe it's best to leave it as is.

- 6. It is not clear how much work the manual curators did for the manual annotation. (The time they spent is also results.) Since the motivation is automation and saving time, more information on the time would be beneficial.

  Our Response: We have this documentation so we revised the text in the methods to include the hours spent on curating the training set. We appreciate the reviewer's attention to such details throughout.

- 7. Please add percentages with the fractions. Also put the resulting numbers in parentheses without explaining it is F1 score). This will get rid of a lot of unnecessary text.

  Our Response: We have revised to add percentages and reviewed the text throughout. Note that because we chose to use precision in for classification, we were deliberately explicit in which metric was used in an effort to keep it clear for readers.

- *8. Figures:*
  a. Color does not show up with black and white printing. Check how it looks in black and white.
  b. Figure 1 is blurry.
  c. Figure 3 is a little confusing going bottom to top. I think it would be less confusing flipping it from top to bottom. It is also not clear what classification layer was used for the algorithms.
  d. Figure 7 and 8 are hard to read in black and white printing.

  Our Response: Thank you for pointing out the issues with our figures. Figure 1 has been re-exported to ensure image quality. For Figure 3, the bottom-to-top orientation isn't unusual to see (e.g., Devlin et al., 2019 https://doi.org/10.48550/arXiv.1810.04805), and it's our own preference so we prefer to leave it as is. Regarding the classification layer, the text is more explicit by stating that a linear classification layer is used. We believe this figure borders on too complex already, so we prefer to leave it as is. Figure 7 has been modified such that the land-masses are white and the borders are drawn in black. We believe this improves contrast and readability in black and white. We have added a patterned fill to figure 8 to improve readability in black and white.

- *9. How many articles were reviewed in the 10% sample (page 25). It is hard to understand with the denominator of 468. It seems that 18 were reviewed (13+5).*

  Our Response: All 468 were reviewed. We have revised the text in that section to clarify.

Exceptional:

1. The open science implementation plan is amazing! I like that the authors are implementing what they believe in.

Our Response: We are very gratified to see acknowledgement of our open science efforts. Thank you for this!

2. It is great that the authors deduplicate articles but still save all articles. Maybe both the original article and the most recent one could be saved?!

Our Response: We don't quite understand this comment. Do you mean drop all those between the first and the most recent? We saved all for the sake of replication and being able aggregate metrics (e.g., all citations, etc.).

3. Thank you for disclosing the data issues faced.

Our Response: Thank you for this as well. We were uncertain what to do at the time, and it's validating to see transparency valued.

Reviewer #3:

The authors of the submitted manuscript report on a thorough work on producing an open catalogue of biodata resources. They describe the process of collecting and verifying information for such a catalogue. The authors used ML, NLP plus manual adjustments. The code and the catalogue itself are made available to the research community. While the used methods are straight forward, their usage is well justified and applying them to a large corpus of scientific publication is a time-consuming process. The manuscript is well written and structured, the reported results are of great importance. Therefore, I believe this manuscript should be accepted for a publication after minor revisions.

Our Response: It was gratifying to see these very positive comments!

- Suggestions for improvement of the current draft: [133 'used a machine-learning based approach' It is not clear at the beginning of the manuscript what ml was employed? Is it using BERT? It should be explained from the beginning.

  Our Response: We have revised the text to introduce our use of BERT much earlier. Thank you for catching this omission.

- In my view, the manuscript could be shortened. For example, there is a bit of repetition in sections 2 and 3. Also, some parts could be moved to SI (supplementary information).

  Our Response: We did revise throughout to tighten the text and remove as much duplication as possible. It is not shorter due to suggestions from other reviewers; however, their suggestions were valuable and we think the additions strengthen the paper even whilst lengthening it.

- [555] '111 resources were funded by international agencies...' It is a lot of resources. It is not clear why funding agencies that could not be mapped to a single country were not shown.

Our Response: We extracted funding information for 1714 resources so that's 111/1714 (6.5%), which is not trivial but relatively small. We're not exactly sure how we would have mapped the multinational funders. For example, if we had tried to map all the individual countries associated with the EU, would we have included or excluded the UK as funding was both before and after Brexit? Or did the reviewer mean something else?

- [623] 'the majority of life science resources within both re3data (975/1189, 80.5%) and FAIRsharing 635 (1161/1640, 70.8%) are not found in our inventory'. That is a real worry. This warrants a more detailed investigation, explanation and suggestion for a resolution.

  Our Response: We absolutely agree that a more detailed investigation is warranted. We know from the preliminary work presented here, however, that this will certainly require a substantial amount of human curation and would constitute another study entirely. Our preliminary analysis suggests that none are entire subsets of each other, but without more careful investigation we don't know the true extent of the overlap (or lack thereof), so we have edited the text to be less premature in our conclusions. Regardless, our initial results are surprising enough to justify the follow-up work required, and we're glad there's interest in seeing it!

- Personally, I do not like the title: 'A machine learning-enabled open biodata resource inventory from the scientific literature'. I suggest or to be more specific what ml was used, or drop mentioning of ml. I do not see why it is so important that ml was used to make it to the title. The important aspects are: open, re-usable, updatable, scientific literature driven.

  Our Response: We were very gratified to see the reviewers' strong appreciation for open science! Thank you! In regards to dropping ML from the title, we'd like to respectfully decline as ML was the main methodical approach so it seems important to forefront. We also think it would be good to keep the ML as the broad term to pique the interest of ML researchers and reinforce ML's utility to life sciences readers.

Just to add, it was interesting to see the results of Mid-Project evaluation and iteration.

Our Response: Thank you!

---

6. PLOS authors have the option to publish the peer review history of their article (what does this mean?).

Reviewer #1: No
Reviewer #2: No
Reviewer #3: **Yes:** Larisa Soldatova

Our Response: We respect the reviewers' choices, although we'd just like to say that it's too bad that Reviewer 2 especially didn't opt to be identified because they provided a very high quality review.
