# Supplementary material for: A machine learning-enabled open biodata resource inventory from the scientific literature
Source: PLoS One. 2023 Nov 28;18(11):e0294812. doi: 10.1371/journal.pone.0294812 (PMC10684096; doi:10.1371/journal.pone.0294812)
Supplement: Attachment — Submitted filename: PONE-D-23-16263 Second Response to Reviewers.pdf [file pone.0294812.s013.pdf]

November 3, 2023

Subject: Second Response to Reviewers for Manuscript No. PONE-D-23-16263

Dear Editor Bernasconi,

We were very glad to read that our first revision of our paper largely satisfied the reviewers' comments! Their suggestions improved our manuscript, and we appreciate their time and interest in our work. For the additional points offered by Reviewer 2, we provide inline responses to those comments as done before, indicating where changes have been made to the revised manuscript or our rationale when we declined to make changes.

Reviewer Prompts/Comments

1. If the authors have adequately addressed your comments raised in a previous round of review and you feel that this manuscript is now acceptable for publication, you may indicate that here to bypass the "Comments to the Author" section, enter your conflict of interest statement in the "Confidential to Editor" section, and submit your "Accept" recommendation.

    Reviewer #1: All comments have been addressed
    Reviewer #2: (No Response)
    Reviewer #3: All comments have been addressed

Our Response: No action needed.

2. Is the manuscript technically sound, and do the data support the conclusions?

    Reviewer #1: Yes
    Reviewer #2: Yes
    Reviewer #3: Yes

Our Response: No action needed.

3. Has the statistical analysis been performed appropriately and rigorously?

    Reviewer #1: Yes
    Reviewer #2: N/A
    Reviewer #3: Yes

Our Response: No action needed.

4. Have the authors made all data underlying the findings in their manuscript fully available?

    Reviewer #1: Yes

Reviewer #2: Yes
Reviewer #3: Yes

Our Response: No action needed.

5. Is the manuscript presented in an intelligible fashion and written in standard English?

Reviewer #1: Yes
Reviewer #2: Yes
Reviewer #3: Yes

Our Response: No action needed.

6. Review Comments to the Author

Reviewer #1

Thank you for addressing the different suggestions and comments. The manuscript has greatly improved and is very much worth publishing.

Our Response: We are happy to hear so!

Reviewer #2:

I want to thank the reviewers for including my comments in their work. I do agree that the manuscript is more readable overall. In general, it seems the biggest difficulty with this paper is delineating the methods and the results section in part because of the mid-project evaluation. I do think the current delineation is much better and I have a few minor comments and places that can be cut out due to repetition to offer.

Our Response: This is great for us to hear. Specific points are addressed below.

Minor comments (in order of appearance):

1. I am surprised by how short the training set curation took. That is great. It seems that the task was pretty simple for them to do which you may want to comment on in relation to the portion that gets manually reviewed based on the threshold for prediction. The idea being that manual review is quick and easy even if it needs to happen for almost half of them.

Our Response: Yes, relatively speaking, we found curation to be fairly straightforward, which did make the mediated approach feasible. We revised the text to make this point while presenting our rationale for doing the selective mediated review.

2. Section 3.3.1 (page 28): the paragraph after figure 6 is too long and mostly repeating the methods about the mid-project evaluation. The results are that the

method chosen was the selectively mediated approach, the threshold of 0.978, and the sentences starting with "In the 468 articles from the 10% sample..." Potentially this paragraph should start with the last sentence because that sums up the main results for this section.

Our Response: We tried to revise as suggested, but the text was cumbersome when placed elsewhere, in particular because it does contain results (e.g., precision vs F1 lowered recall to an unacceptable level for NER). This caused the text to be quite disjointed, so we respectfully decline to implement this suggestion.

3. Section 3.3.1 (page 29): similarly to comment 2, the next paragraph starting with "During preparation of this manuscript, we realized..." is methods. The paragraph should start with "In the mid-project evaluation, 7 articles from the classification test and 5 articles from the NER test set were found..."

Our Response: We have revised this paragraph and moved some non-redundant content to methods.

4. Section 3.3.2: There are a lot of records flagged for manual review (almost half if you add up all the different reasons for being flagged. Harkening back to comment 1, I think you can say that manual review is quick and easy so it doesn't matter that it is so much. It is also interesting to note the number that were flagged here vs. the test data if that is easy to do.

Our Response: We added this point explicitly in the method sections already per suggestions above, so we respectfully decline as other suggested revisions asked us to remove such explanatory text from the results sections.

5. In general section 3.5 is a great example of just results and pretty short. I would still say that you can cut out part of the first sentence of section 3.5 starting with "After curation and processing to remove..." Instead the section can start with "The resulting inventory contained 3112 resources..."

Our Response: We respectfully decline this suggestion as we believe the opening clause helps keep the reader oriented.

6. Section 3.5.2: The first paragraph talks about the metadata and everything after "Both PubMed Central and Europe PMC, which exchange data,..." is introduction or discussion/future work. I don't think it belongs in results. Further the last paragraph in that section is also discussion/future work starting with "The articles associated with biodata resources within the inventory also provide opportunities to glean...". This whole paragraph is mostly stated as future work. If you want it to be results about the articles instead, then maybe providing an example of why they are useful or could be used would be helpful.

Our Response: Only the last half of the last sentence in this paragraph has anything to do with future work, and even that was in direct relation to the results. Our point about the irregular reporting of smaller funders (especially those outside of the US and UK) directly precedes our results where we found increasingly more granular funding sources reported. There is no good place in the introduction or the discussion to bring this up and even if so, it would be too far away from where this context is most helpful for the reader. We have edited slightly, however, to make it more clear that we see this directly in the results of our work.

7. In the discussion section, the second paragraph states, "the selected article classification model achieved..." Please state the name of the models that performed the best for classification and NER.

Our Response: We have edited to explicitly state the model names in the discussion as well.

8. In the discussion section, the added discussion on why the overlap between resources is low is great. One comment mentioned is that the selection criteria may differ in subtle ways. I guess can you give examples or do a very basic analysis of the differences?

Our Response: We have edited, and provide an example we point out that that definition of “data” itself is not universally shared.

9. In the discussion section (page 39). The sentence starting with "One potential use of the inventory as a catalyst for outreach." needs to start with "This is one potential use of the inventory..."

Our Response: We have corrected the typo. Thank you for catching it.

Response to authors comments on my review:

1. I see that you are using the validation set to choose the algorithm and then using the test set to report on unseen data. Thank you for the explanation.

Our Response: You’re welcome.

2.Thank you for your comment about the "true negatives". It is very interesting to think about in theory vs. practice.

Our Response: You’re welcome.

3. I hear the point about the manuscript being very long. I think mentioning more

things in future work is totally fine. I do believe there is good reception of this work and that it is interesting.

Our Response: Great to hear!

4. I understand the authors' decision to keep the structure following PLOS One guidelines. Overall the structure is much clearer than before.

Our Response: Great to hear!

5. Minor revision comment 2: I was asking about the latter option of whether knowledge-bases and ontologies were included in your definition and find that interesting in general and maybe it should be mentioned as well. I like the discussion in the paper about how to define a biodata resource.

Our Response: We did not restrict our definition to only experimental data, so knowledge bases and ontologies could also meet the criteria and, in fact, did. Two examples of ontologies in the inventory are FOAM (Functional Ontology Assignments for Metagenomes) and ENVO (Environmental Ontology), while two examples of KBs in the inventory are FROG-kb (Forensic Resource/Reference on Genetics-knowledge base) and GPKB (Genomic and Proteomic Knowledge Base). There may be others as well but we provide these just as examples to know that ontologies and knowledge bases are included.

6. I do understand the difficulty with methods and results so take my comments around that with a grain of salt.

Our Response: Excellent. Thank you!

7. Adding F1 score makes sense when you do have two different metrics you use. Thank you for the response.

Our Response: You're welcome.

8. Thank you for the figure comments. All of that makes sense.

Our Response: Great!

9. In terms of the deduplication comment I made in the exceptional category, I see that you do keep the articles so you are all good.

Our Response: Great! Thank you again for your time and terrific attention to detail.

Reviewer #3

I am satisfied with the revisions made. The authors have addressed the comments and suggestions by other reviewers reasonably well.

Our Response: We are very glad to hear so!

7. PLOS authors have the option to publish the peer review history of their article (what does this mean?). If published, this will include your full peer review and any attached files.

Our Response: Great to have 2 of 3 allow their names open! We appreciate all of their time and very helpful comments.
